# Zinc Phthalocyanine Sensing Mechanism Quantification for Potential Application in Chemical Warfare Agent Detectors

**DOI:** 10.3390/s22249947

**Published:** 2022-12-16

**Authors:** Paulina Powroźnik, Barbara Solecka, Piotr Pander, Wiesław Jakubik, Fernando B. Dias, Maciej Krzywiecki

**Affiliations:** 1 Institute of Physics—Center for Science and Education, Silesian University of Technology, S. Konarskiego Str. 22B, 44-100 Gliwice, Poland; 2 Faculty of Chemistry, Silesian University of Technology, M. Strzody 9, 44-100 Gliwice, Poland; 3 Department of Physics, Durham University, South Road, Durham DH1 3LE, UK

**Keywords:** zinc phthalocyanine, DMMP, thermal desorption spectroscopy, adsorption energy, desorption activation energy, sensing mechanism, chemical warfare counteraction, impedance spectroscopy

## Abstract

Rapid and accurate detection of lethal volatile compounds is an emerging requirement to ensure the security of the current and future society. Since the threats are becoming more complex, the assurance of future sensing devices’ performance can be obtained solely based on a thorough fundamental approach, by utilizing physics and chemistry together. In this work, we have applied thermal desorption spectroscopy (TDS) to study dimethyl methylophosphate (DMMP, sarin analogue) adsorption on zinc phthalocyanine (ZnPc), aiming to achieve the quantification of the sensing mechanism. Furthermore, we utilize a novel approach to TDS that involves quantum chemistry calculations for the determination of desorption activation energies. As a result, we have provided a comprehensive description of DMMP desorption processes from ZnPc, which is the basis for successful future applications of sarin ZnPc-based sensors. Finally, we have verified the sensing capability of the studied material at room temperature using impedance spectroscopy and took the final steps towards demonstrating ZnPc as a promising sarin sensor candidate.

## 1. Introduction

Sensing of complex gas molecules is becoming a critical issue, not only for the purpose of environmental monitoring, but also due to increasing threats of terrorist activities that involve the use of chemical weapons, such as sarin. One of the possible “passive” counteractions is the development of fast-acting and reliable sensor devices that could warn users upon sarin detection. In real life applications, these sensing devices are exposed to a wide range of interferences, such as parasitic effects, time- and environment-related degradation effects, and many others. Therefore, a crucial property of a sensor is its effectiveness and ultimate performance, despite the presence of these detrimental interferences. There are many components that impact the performance of sensors and some of the most important components are as follows: (1) designation of an appropriate sensing material, (2) suitable construction of the sensor device, and (3) optimization of the sensing (adsorption) mechanism towards a more effective interaction between a particular analyte and the active material. However, it is clear that among these three, the sensing mechanism is the most important parameter. Hence, understanding the sensing mechanism and its successful use to achieve a desired effect is paramount for the successful development of sensor devices.

R. W. Catterall defined a chemical sensor as a device that responds to a particular analyte in a selective way through a chemical reaction that can be used for the qualitative or quantitative determination of the analyte [1]. Using this definition, one can note that the effectiveness of the sensor is closely linked to (i) the properties of the sensing material and (ii) the effective use of the material, including analyte interactions. Several types of sensors can be designed depending on the sensing mechanism at work, namely catalytic sensors, electrochemical sensors or solid state-based sensors. Although all the above-mentioned types of sensors are already in use nowadays, the third group is a rapidly emerging branch of the sensor industry. One of the most popular and reliable groups of solid-state sensors are resistive sensors, which are based on organic molecules from the group of phthalocyanines [2,3,4,5,6,7]. In these devices, the sensing effect is highly related to the gas sensing material, since their electronic properties are strongly affected by the adsorption of foreign molecules [8,9,10]. Although phthalocyanines have been considered as a promising material for gas sensing for many years [11,12,13], the actual sensing mechanism has not been fully characterized.

One of the most important parameters in the evaluation of the sensing mechanism is the adsorption energy (*E_ads_*). *E_ads_* has a direct impact on the sensitivity to the specific gas, since it is correlated with the amount of charge transfer between the gas molecule and the sensing material’s surface. Thus, *E_ads_* is the first parameter that determines whether a material can be used as a sensor for the gas of interest. *E_ads_* can be estimated experimentally from desorption studies by measuring the desorption activation energy (desorption without decomposition) [14]. Another issue to consider while evaluating the sensing mechanism of complex gas molecules is their desorption and decomposition on the sensing material’s surface. This is especially important for the sensor’s recovery. If one aims to design a reusable sensor, desorption and decomposition mechanisms should be investigated as a priority. The desorption temperature is also a crucial parameter that indicates the recovery temperature of the sensor, which must be below the decomposition temperature of the sensing material. The study of the decomposition of gas molecules upon desorption provides crucial information about new substances that are released into the environment, some of which may have the potential to be re-adsorbed and impact the surface of the sensing material.

To keep the experiments as safe as possible, dimethyl methylphosphonate (DMMP) was used as a sarin simulant. Desorption and decomposition of DMMP have been studied in the past, but more recent works on this subject can also be found [15,16,17,18,19,20,21,22,23,24,25,26,27,28,29,30,31]. However, most of the works published so far have been focused specifically on applications regarding molecule disintegration rather than on the context of sensing. Decomposition is indeed rarely considered in the context of gas sensors, and most studies are focused on metal oxides. Therefore, there is clearly a lack of studies concerning the DMMP decomposition on organic sensing materials, such as phthalocyanines. Following our previous work [32], where photoemission spectroscopies and theoretical modeling were combined, we decided to take a step forward. In the current work, we aim to achieve a better understanding of the gas–surface interactions by using adsorption energy as a fundamental factor behind the adsorption phenomenon. For this reason, we propose a research protocol that combines high-end simulations with thermal desorption spectroscopy (TDS) and impedance spectroscopy. We use impedance spectroscopy for both qualitative and quantitative adsorption studies, which are fundamental factors in the description of the sensing mechanism. In our work, we use a zinc phthalocyanine (ZnPc) layer deposited on molybdenum (VI) oxide (MoO_3_) as a potential sensing material. The choice of ZnPc was based on our previous results of DMMP and sarin interactions with methallophthalocyanines (MPcs) [32]. The MoO_3_ layer serves as a substrate for ZnPc deposition.

## 2. Materials and Methods

### 2.1. Deposition of Zinc Phthalocyanine Layers

Zinc phthalocyanine (ZnPc, 97%, Sigma Aldrich, Saint Luis, MO, USA) (10 nm) was deposited by vacuum thermal evaporation onto glass substrates with interdigitated gold electrodes (Metrohm DropSens) and molybdenum (VI) oxide (MoO_3_) in a thin layer (10 nm). MoO_3_ served as a substrate for the ZnPc thin-layer uniform deposition on the glass substrates with gold electrodes. The substrates were pre-cleaned with isopropanol and dried with pure N_2_, then UV–ozone cleaned for 5 min. Prior to ZnPc deposition, a layer of MoO_3_ (MoO_3_ 3-6 mm pieces, 99.95%, Testbourne Ltd., Basingstoke, UK) was evaporated on the substrate at room temperature in high vacuum conditions, using the Lesker Spectros II Evaporation System with quartz crystal microbalance (QCM) thickness control. The base pressure was 10^−6^ mbar. The deposition rate was kept at the level of 0.1–0.3 Å/s. Reference samples coated with MoO_3_ were removed, while the remaining MoO_3_-coated substrates were subsequently coated with a 10 nm thick layer of ZnPc. For ZnPc, the deposition rate was kept at 0.5 Å/s.

### 2.2. Exposure to DMMP

To keep the experiments as safe as possible, we used dimethyl methylphosphonate (DMMP) as a sarin experimental equivalent. The usage of DMMP was rationalized by the following: (i) similarity of its chemical structure to sarin (especially the presence of the P=O bond that plays a crucial role in sarin sensing) and (ii) the negligible difference in the adsorption energy on metal phthalocyanines (MPcs) between sarin and DMMP, as demonstrated previously [32]. For TDS experiments, DMMP exposure was carried out using the Owlsone Inc. vapor generator (OVG-4). Samples were placed in the environmental cell that contained a gas inlet and outlet. The DMMP vapor was obtained from a certified permeation tube (Owlstone Inc., Westport, CT, USA). Nitrogen 5.0 (Air Liquide) was used as a carrier gas. Details of the procedure can be found in an earlier work [32].

### 2.3. Thermal Desorption Spectroscopy Experiment

The TDS experiments were performed with bare MoO_3_ and MoO_3_ coated with ZnPc, before and after exposure to DMMP, in order to identify the desorbing ambient-related and DMMP-related species. Thermal desorption on the surfaces was performed by thermal annealing with a controlled temperature ramp (PID-monitored power supply, heating rate of 0.5 K/min; base pressure of 7.5 × 10^−10^ mbar). Partial pressure of the selected desorbing species was controlled with an RGA 100 (Stanford Research Systems) quadrupole mass filter in the temperature range of 515–675 K. The experimental setup was limited to detecting species with an atomic mass of up to 100 amu and to simultaneously controlling up to 10 different species. We monitored the following masses in order to detect the expected products of DMMP decomposition [26,27,28,29,30,31,32,33] and the species adsorbed from the atmosphere: 2 amu (H_2_), 18 amu (H_2_O), 28 amu (CO/N_2_), 44 amu (CO_2_/ H-C≡P), 31 amu (P), 79 (PO_3_), 94 amu (methyl phosphonate, CH_3_PO_3_), 46 amu (dimethyl ether, Et_2_O), 15 amu (CH_3_), and 30 amu (formaldehyde, CH_2_O). The spectra were decomposed using Fityk software (1.3.1 version) [33].

### 2.4. Characterization of Sample Topography

Studies using atomic force microscopy (AFM) were conducted with a non-contact PSIA XE-70 microscope fitted with Budget Sensors silicon probes (TAP-300AL-G cantilevers, 300 kHz resonance frequency; spring constant of 40 Nm^−1^). We used Gwyddion^®^ software to correct sample inclination and distortions caused by the z-scanning stage [34].

### 2.5. Computational Modeling

**Figure 1:**
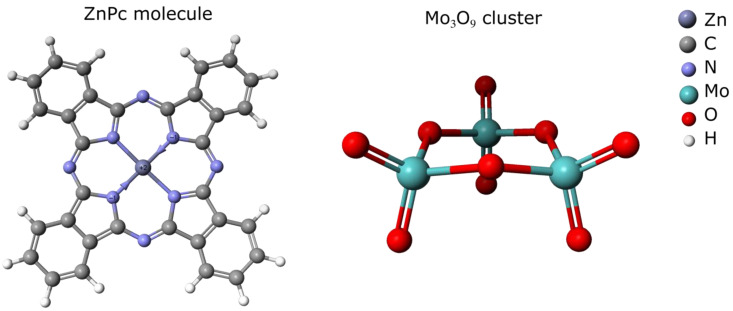
ZnPc molecule and MoO_3_ cluster used in semi-empirical computations.

All semi-empirical calculations were performed using the PM6 method with the MO-G simulation in the SCIGRESS software package (version FJ 2.8 EU 3.2.2.). The simulations were carried out for a single molecule of ZnPc and a Mo_3_O_9_ cluster (Figure 1) with one molecule of analyte gas. The adsorption energies on ZnPc and MoO_3_ for each analyte gas were determined from Equation (1).

(1) $$E_{ads}=E_{str+gas}-(E_{str}+E_{gas})$$

where *E_ads_* is th adsorption energy in eV, *E_str_* is the ground state energy of the adsorbent structure (ZnPc or MoO_3_) in eV, *E_gas_* is the ground state energy of the analyte gas molecule in eV, and *E_str+gas_* is the ground state energy of the global minimum of the adsorbent/analyte gas structure in eV.

### 2.6. Sensor Response Measurements Using Impedance Spectroscopy

**Figure 2:**
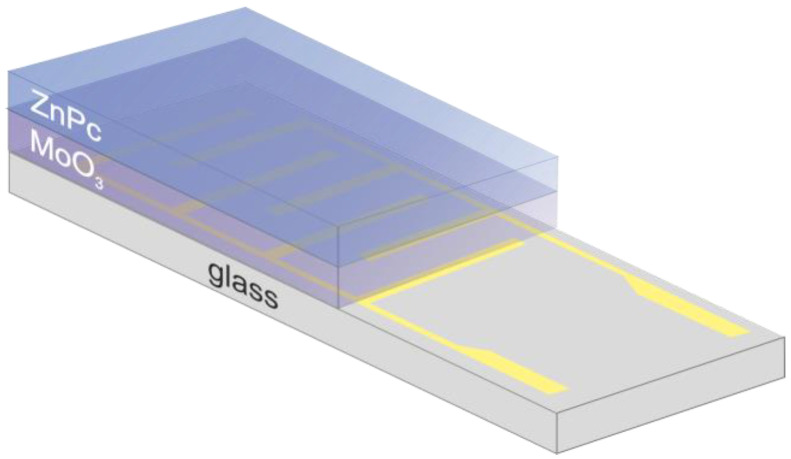
The diagram of the sensing structure: the thin layer of ZnPc layer deposited on the glass substrate with gold electrodes, coated with an MoO_3_ layer.

The sample deposited on the glass substrate with inter-digited electrodes (Figure 2) as described above was placed in the environmental cell that contained a gas inlet and outlet and electrical feedthrough. DMMP vapors were prepared as described in Section 2.2, using synthetic dry air (~5% relative humidity, Air Liquide) as a carrier gas. The impedance spectrum was recorded in the frequency range of 40 Hz–1 MHz for a sample exposed for 5 min to pure air with different flow rates (500 mL/min, 400 mL/min, 300 mL/min, and 200 mL/min). Similar measurements were carried out for air flowing through the permeation tube with DMMP. After each exposure to DMMP, the sample was regenerated in the flow of pure air (500 mL/min). The following DMMP concentrations were obtained: 270 ppb, 340 ppb, 450 ppb, and 675 ppb, respectively, for the flow rates listed above. All measurements were performed in a planar mode with a two-probe method, using the Agilent 4294A Precision Impedance Analyzer.

## 3. Results and Discussion

### 3.1. Thermal Desorption—Survey Approach

**Figure 3:**
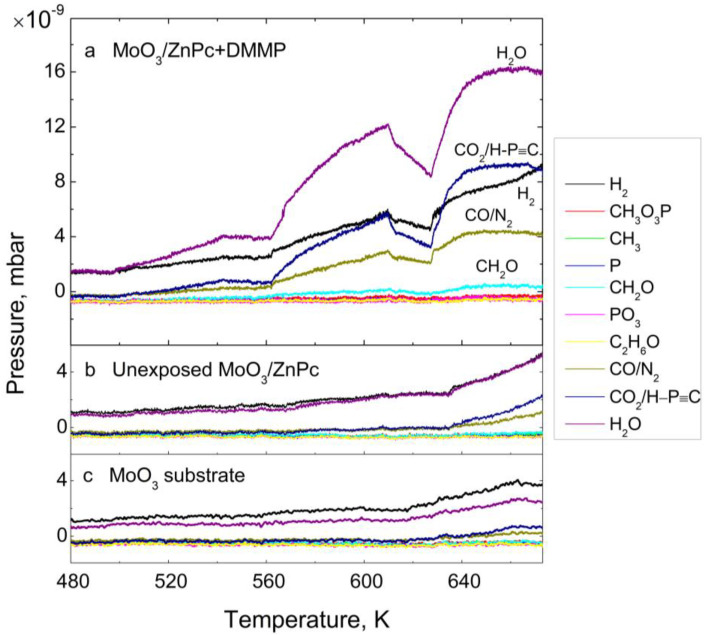
The survey TDS spectra for (**a**): the MoO_3_/ZnPc structure after DMMP exposure; (**b**): unexposed MoO_3_/ZnPc structure and (**c**): bare MoO_3_.

**Figure 4:**
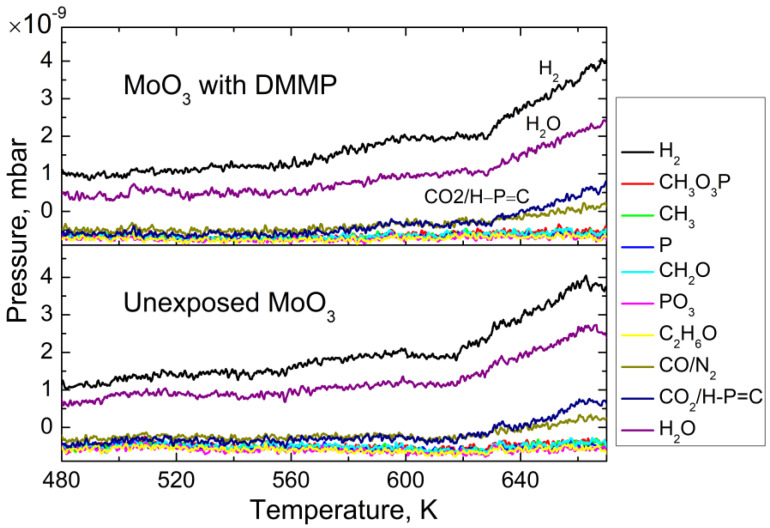
The survey TDS spectra for the MoO_3_ substrate after DMMP exposure and unexposed MoO_3_ substrate.

The survey TDS spectra for DMMP-exposed MoO_3_/ZnPc, unexposed MoO_3_/ZnPc and bare MoO_3_ structures are presented in Figure 3a–c, respectively. Figure 3 shows the spectra of the DMMP-exposed MoO_3_ substrate in comparison with unexposed MoO_3_. The traces are in both cases identical; thus, we can assume that the desorption products from the MoO_3_ substrate do not include the degradation products of DMMP. As DMMP desorption from the ZnPc layer is crucial for this study, the discussion will be focused on the spectra of the DMMP-exposed MoO_3_/ZnPc structure. In Figure 3a, one can note that among the monitored atomic masses, the desorption of particles with masses of 2, 18, 28, 31, and 44 amu was observed at temperatures above 500 K. Importantly, only one of the desorbing species can be clearly assigned as a DMMP decomposition product—30 amu, formaldehyde (CH_2_O). All the other species desorb from the unexposed MoO_3_/ZnPc structure, as well as from the bare MoO_3_ substrate (Figure 4b,c). The atomic masses of 2, 18, 28, and 44 amu can be assigned to the air-related impurities, such as H_2_, H_2_O, CO/N_2_, and CO_2_, respectively. However, the partial pressures of those species that desorbed from the DMMP-exposed sample are 2–4-fold higher than in the case of the unexposed sample. Significantly, the character of the desorption trace is different for the DMMP-exposed sample than for the one that remain unexposed, indicating a more complicated nature of the desorption process in the presence of adsorbed DMMP on the ZnPc surface. The following DMMP decomposition products were identified: H_2,_ H_2_O, CO and CH_2_O, which could be identified and assigned accordingly, and the mass of 44 amu may be assigned either to CO_2_, H-C≡P or to both of these species that desorb together.

**Figure 5:**
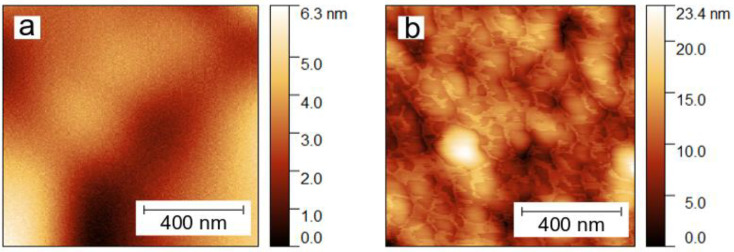
The AFM topography images (1 µm × 1 µm) of (**a**): the bare MoO_3_ layer, and (**b**): MoO_3_/ZnPc bilayer.

As demonstrated previously using DFT calculations [32], the highly ordered thin ZnPc layers are required to enhance the sensitivity to DMMP by increasing the availability of preferred adsorption sites for covalent bonding (i.e., Zn atoms). Therefore, we used AFM to study the ordering of the ZnPc layer surface (Figure 5). We found a moderately developed surface of the MoO_3_ layer, with its roughness increasing by ~5-fold after deposition of ZnPc, leading to a considerable development of its surface. The latter is closely linked to larger quantities of available adsorption sites. Since the obtained ZnPc layers are disordered, one can expect a significant contribution of vdW interactions with DMMP molecules. However, we also expect a certain quantity of adsorption sites to be available for covalent bonding between Zn atoms and DMMP, which will be relevant in further analysis.

### 3.2. Thermal Desorption—Components Analysis

In a more detailed analysis of the TDS spectra, we observed that the recorded signal that represents the partial pressures of the investigated species underwent a single-component detection procedure. For this purpose, the detected TDS traces relevant to the given molecular species were decomposed by applying a peak-fitting procedure, using an asymmetrical Gaussian function that is applicable for first-order desorption kinetics. We assumed first-order kinetics following the work of Contour et al. who studied desorption from phthalocyanines [35].

**Figure 6:**
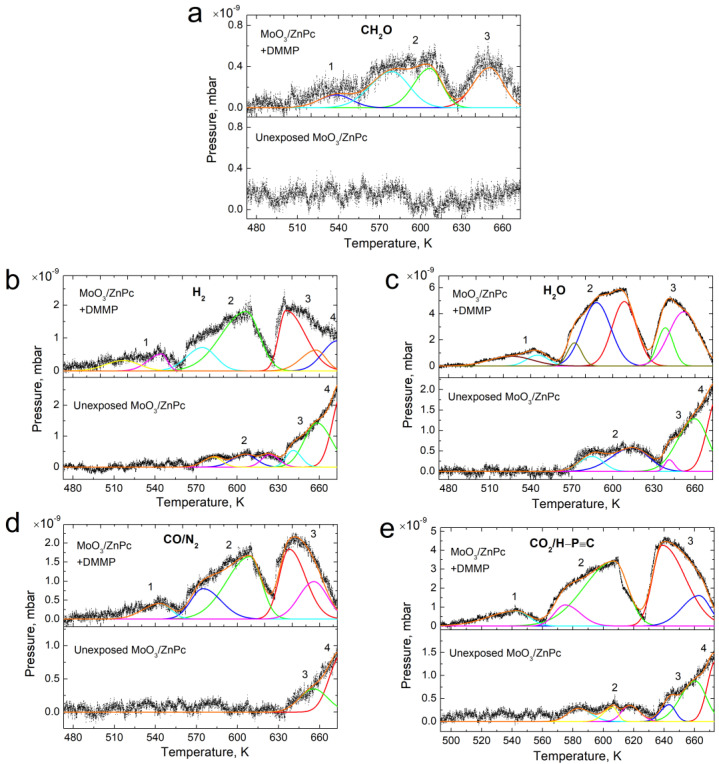
The decomposition traces (for the desorption windows) of unexposed MoO_3_/ZnPc and DMMP-exposed MoO_3_/ZnPc structures for the following species: (**a**) CH_2_O (30 amu)), (**b**) H_2_ (2 amu), (**c**) H_2_O (18 amu), (**d**) CO/N_2_ (28 amu) and (**e**) CO_2_/ H-C≡P (44 amu).

The decomposed traces for all the considered molecular species in unexposed and DMMP-exposed MoO_3_/ZnPc samples are presented in Figure 6. For the DMMP-exposed sample, we present differential traces (i.e., the signal from an unexposed sample is used as a baseline and subtracted from each trace). Components in the analyzed traces were divided into four groups (namely 1–4), according to the following peak temperatures: 1 (<570 K), 2 (570–610 K), 3 (635–670 K) and 4 (>670 K), which correspond to different binding sites. For each peak, the activation energy of desorption was calculated by applying the Redhead formula for first-order desorption kinetics [36].

(2) $$E_{des}=RT_{max}\left[\ln\left(\frac{vT_{max}}{\beta }\right)-3.46\right]$$

where

*R*—gas constant, 8.314 J mol^−1^ K^−1^;
*ν*—pre-exponential factor, s^−1^;
*T_max_*—temperature at peak, K;
*β*—heating rate, K s^−1^.

Redhead’s model of desorption was applied as a common approach to estimate the activation energy for desorption from a single trace for a single DMMP exposure [37]. The surface coverage is defined as the number of adsorbed molecules on a surface divided by the number of molecules in a filled monolayer of that surface [38]. The value of the pre-exponential factor *ν* is often assumed to be equal to 10^13^ 1/s. For this value of *ν* and *β* = 0.5 K/min, the condition for the use of Redhead’s formula (*ν/β* between 10^8^ K^−1^ and 10^18^ K^−1^) is satisfied (*ν/β* = 10^15^ K^−1^). All the estimated peak temperatures with corresponding *E_des_* are shown in Table 1. Additionally, we calculated the area under each Gaussian curve, as they are proportional to the surface coverage [39]. The relative area for each desorbing species as a fraction of the sum of all desorbing components is also presented in Table 1. Desorption of formaldehyde (30 amu) (Figure 2a) from the DMMP-exposed sample starts at around 510 K, with a small and broad signal (group 1), reaching its maximum at 538 K. It is then followed by four larger peaks assigned to the group 2 and 3. The *E_des_* of four different DMMP-related formaldehyde binding sites ranged from 1.7 eV to 2.1 eV (Table 1). The different energy states may be a result of desorption from different depths of the adsorbing layer. Since the ZnPc sample exhibits a highly developed surface (as confirmed by the AFM results above), part of the DMMP molecules are likely to be directly adsorbed on the surface, while others penetrate deeper into the film through structural defects. One must also keep in mind that the studied ZnPc layer is disordered; therefore, different adsorption geometries will be present depending on the availability of preferable adsorption sites. The relatively low partial pressure of the desorbing formaldehyde is most likely due to only part of the adsorbed DMMP being covalently bonded to the ZnPc surface and decomposing in the given range of temperatures. Other DMMP molecules were likely to be attached by weak vdW forces and desorbed without decomposition at a lower temperature. This is consistent with our previous findings [32], which showed that DMMP preferably forms covalent bonds with the MPc central atom. When the MPc layer is not perfectly ordered, the preferable adsorption sites are limited and different adsorption geometries with lower binding energies can be formed. For vertically stacked Pc molecules, DMMP can attach to the surface only through vdW interactions.

The differential traces of all the other formaldehyde species follow, to some extent, the structure of the formaldehyde desorption trace. All of them reveal the first low-intensity peak with a maximum value around 540 K, followed by two and three energy states. In the case of hydrogen (Figure 6b), we observe a desorption component attributed to group 4 of signals, which can be assigned to the partial decomposition of the ZnPc molecule [40].

### 3.3. Semi-Empirical Modeling of Adsorption Energies

**Figure 7:**
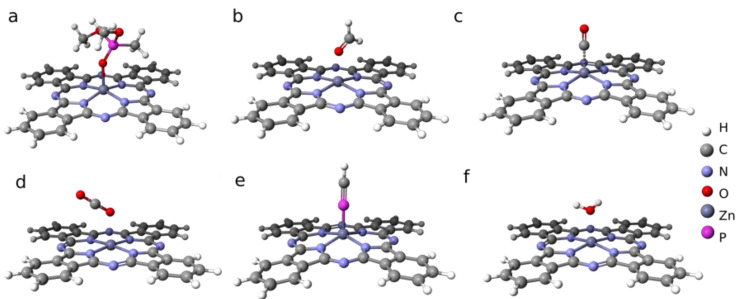
Geometries of ZnPC adducts with: (**a**) DMMP, (**b**) CH_2_O, (**c**) CO, (**d**) CO_2_, (**e**) PHC, and (**f**) H_2_O.

**Figure 8:**
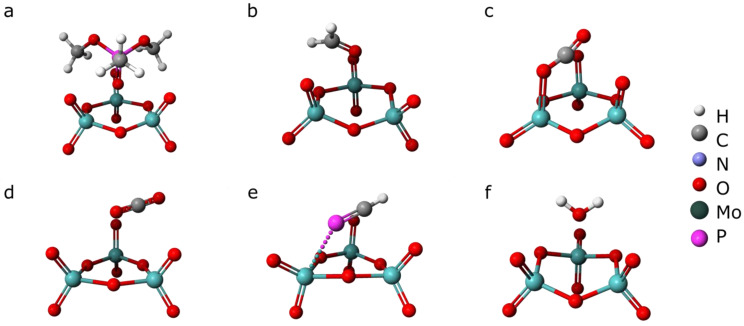
Geometries of MoO_3_ adducts with: (**a**) DMMP, (**b**) CH_2_O, (**c**) CO, (**d**) CO_2_, (**e**) PHC, and (**f**) H_2_O.

In order to compare the desorption activation energies with theoretical adsorption energies for all detected species, the semi-empirical modeling of DMMP, CH_2_O, CO, CO_2_, H-C≡P, and H_2_O was performed. The calculations were performed for a single molecule of ZnPc and a Mo_3_O_9_ cluster with a single molecule of each analyte gas. The theoretical modeling of transition-metal systems requires a high level of computation [41]. The cluster approach used in this work can be applied to molybdenium(VI) oxide, since the cluster represents the surface with sufficient accuracy for our purposes. The calculated adsorption energies are presented in Table 2, together with the minimum activation energies for desorption estimated from TDS. The corresponding geometries of the ZnPc molecule adducts are shown in Figure 7 and Figure 8.

The theoretical adsorption energies of all the analyzed species vary from 0.1 eV up to 1.9 eV, while the experimental desorption activation energy is in the narrow range of 1.8–2.2 eV. This suggests that the desorbing molecules are most likely to be a result of DMMP decomposition rather than just desorption. The desorption activation energy is two-fold greater than the theoretical DMMP adsorption energy on ZnPc. However, one must keep in mind that Redhead’s method gives only a rough approximation of *E_des_*. Moreover, the theoretical value was obtained for an idealized system of DMMP adsorbed on an isolated ZnPc molecule in a vacuum (Figure 7), while the experimental system was far from these ideal conditions. In addition, the difference between the desorption activation energy for decomposition products and the adsorption energy of DMMP molecules includes the DMMP molecule decomposition energy.

### 3.4. Implications for ZnPc Capability for DMMP Sensing

For a semiconducting material, the change in surface potential (∆*V_S_*) induced by charge accumulation or depletion causes change in energy band bending (*e*∆*V_S_*). According to Morrisson’s equation, the magnitude of changes in the electrical resistance of the sensor (i.e., sensor response) depends on the value of *e*∆*V_s_* [42].

(3) $$e\Delta V_{s}=kT\ln(\frac{R_{g}}{R_{a}})$$

where *e*—elemental charge, e; *k*—Boltzmann constant, eV/K; *T*—temperature, K; *R_g_*—sensor resistance when exposed to analyte gas, Ohm; *R_a_*—sensor resistance in air, Ohm.

From this point of view, high adsorption energy is preferred for enhancing the sensor effectiveness, as for stronger charge transfer (i.e., greater adsorption energy), the value of *e*∆*V_s_* is greater. On the other hand, to obtain a reusable device, the adsorption energy should be low enough to allow for desorption of adsorbed gas at temperatures low enough to prevent degradation of the sensing material and provide reduced power consumption. However, for the sensors of lethal compounds, conditions that ensure good sensitivity and rapid detection are crucial. We estimate the expected sensor responses that result from the change in the band bending to verify if the studied material meets this condition.

The value of *e*∆*V_s_* predicted from modeling can be calculated using the following expression [43]:

(4) $$\Delta \varphi =e\Delta V_{S}+\Delta \chi$$

where ∆*φ*—change in semiconductor work function induced by the adsorption of gas molecules, and ∆*χ*—change in electron affinity caused by the formation of the dipolar layer of the adsorbate.

The value of ∆*φ* (∆*φ* = 0.54 eV) induced by the adsorption of DMMP molecules on a well-ordered ZnPc monolayer was determined earlier [32] using DFT. To calculate ∆*χ,* we used the following expression [44]:

(5) $$\Delta \chi =eV_{dip}=\frac{ep_{n}}{\varepsilon_{0}\varepsilon_{r}\cdot d^{2}}$$

where *V_dip_*—potential difference between the two sides of the adsorbate monolayer, V; *p_n_*—normal dipole moment of the adsorbate layer, C m; *d*—diameter of adsorbed molecules, m^2^; *ε_0_*—vacuum electrical permittivity, F/m; *ε_r_*—relative permittivity of adsorbed molecules.

The value of *p_n_* can be calculated by taking into account the fractional charge transferred upon the bonding of DMMP molecules to the ZnPc species (*q* = 0.3*e*) and bond length (*r* = 2.14 Å) determined in an earlier work [32]. From the formula *p_n_* = *qr,* we obtain *p_n_* = 3.09 D. For DMMP molecules that form the adsorbate layer, we use *ε_r_ =* 22.3 [45] and *d* = 0.58 nm [46], from which we obtain ∆*χ* = 0.15 eV. Finally, by subtracting ∆*χ* from ∆*φ,* we obtain the expected value of *e*∆*V_s_* = 0.4 eV. Since all the calculations were performed for an ideal system where DMMP is adsorbed on a free-standing, well-ordered ZnPc monolayer and all adsorption sites are available, we do not calculate the exact value of electrical resistance change, as it would be highly overestimated. However, from the calculated values, one can note that even if there is a relatively strong dipole formed upon DMMP adsorption on the ZnPc surface, the contribution of band bending to the total change in work function is almost three-fold larger than the change in electron affinity (∆*χ* = 0.15 eV). Thus, we can expect significant changes in electrical resistance (i.e., sensor response). As a result, ZnPc satisfies the first condition for an optimal sensor material. We also find that the temperature for DMMP decomposition and desorption from the ZnPc surface is above 500 K, but is still lower than the decomposition temperature of the ZnPc layer. This allows for the successful recovery of the sensing structure. The second condition for the optimal sensing material is met. The above findings are of vital importance when considering the use of ZnPc in future sensor applications. Yet, our results show the possibility of sensor recovery for DMMP. For sarin, the decomposition products can differ, because of the presence of fluorine atoms in the molecule.

Further information on the sensing capabilities of the examined structures can be derived from the data presented in Table 2 and the desorption traces in Figure 2 and Figure 5. In particular, we were able to obtain information about the availability of adsorption sites, selectivity and reversibility of the adsorption process. The adsorption energies of all the impurities, such as hydrogen, water, and carbon species, on ZnPc are roughly equal or lower than the DMMP adsorption energy. At the same time, the activation energy for the desorption of these analytes from the unexposed MoO_3_/ZnPc structure is higher than for species resulting from DMMP decomposition for the exposed structure. In contrast, the adsorption energies on MoO_3_ are relatively high. This suggests that most of the components that desorb from an unexposed sample are impurities adsorbed from the MoO_3_ substrate. This is consistent with the TDS traces. Even some impurities desorb from ZnPc and the partial pressures of those species resulting from an unexposed sample are much lower than for species resulting from the decomposition of DMMP. Although the studied material can adsorb species from the air, a considerable number of adsorption sites remain available for DMMP. Interaction of the DMMP molecule with the ZnPc surface is stronger and perhaps more specific than with other species that are also likely to be present in air (i.e., H_2_O, CO_2_), which demonstrates ZnPc selectivity to the sarin simulant.

### 3.5. Verification of the Sensing Capability of ZnPc by Impedance Spectroscopy

**Figure 9:**
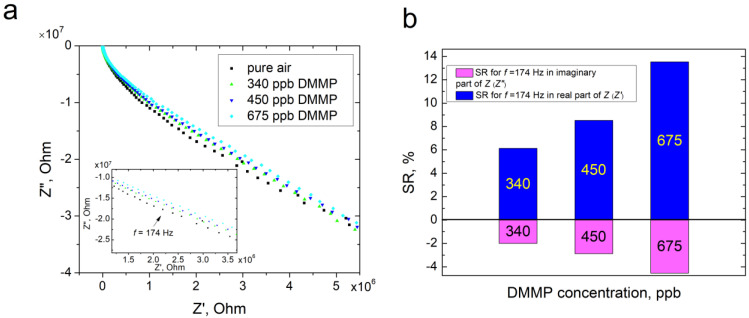
(**a**) Z″(Z′) plot for ZnPc in the frequency range 40 Hz–1 MHz for the sample after exposure to pure synthetic air and subsequent 5 min exposure to the mixture of synthetic air and DMMP at various concentrations; (**b**) sensor responses of ZnPc to DMMP for the real (*Z*′) and imaginary (*Z*″) components of impedance (*Z*).

In order to verify the capability of DMMP sensing with ZnPc by electrical methods, the sensor responses were measured for several DMMP concentrations using impedance spectroscopy. We chose the AC method, since we expected an enhanced sensing effect in respect to DC measurements due to the surface dipole and additional capacitance. Figure 9a presents the *Z*″(*Z*′) (imaginary vs. real impedance components) plot recorded in the frequency range 40 Hz–1MHz for the sample after exposure to pure synthetic air and subsequent 5 min exposure to the mixture of synthetic air and DMMP at various concentrations. Since there were no differences between the impedance spectra for different air flow rates, we present only one characteristic as an example. The lowest concentration for which we have recorded a sensing response is 340 ppb. One can note from Figure 8a that the most significant changes in the *Z*″(*Z*′) characteristics are observed in the frequency range 122–274 Hz. We calculated the sensor responses in the real (*Z*′) and imaginary (*Z*″) components of impedance (*Z*) for a frequency in the middle of this range, f = 174 Hz (Figure 9b), using the following formulas:

(6) $$SR^{\prime }=\frac{{Z^{\prime }}_{DMMP}-{Z^{\prime }}_{air}}{{Z^{\prime }}_{air}}\cdot 100\%$$

(7) $${SR}^{\prime \prime }=\frac{{Z^{\prime \prime }}_{DMMP}-{Z^{\prime \prime }}_{air}}{{Z^{\prime \prime }}_{air}}\cdot 100\%$$

where *SR*′—sensor response in the real component of impedance, %; *SR*″—sensor response in the imaginary component of impedance, %; *Z′_DMMP_*, *Z″_DMMP_*—real and imaginary component of impedance in the presence of DMMP, Ohm; *Z′_air_*, *Z″_air_*—real and imaginary component of impedance in pure air, Ohm.

For both the imaginary and the real component, the sensor response follows the concentration of DMMP in air. However, in the real component of the impedance *Z*, the magnitude is three-fold higher than in the imaginary component. This result is consistent with the theoretical estimation of changes in the ZnPc work function caused by the adsorption of DMMP. From the calculation, we expect a higher value of *e*∆*V_s_* with respect to ∆*χ*. The changes in *Z*′ mean that the electrical resistance of the structure changes due to the adsorption of DMMP molecules, as a consequence of band bending. On the other hand, the alteration of *Z*″ can be assigned to the changes in electrical capacitance, resulting from surface dipole formation [47].

## 4. Conclusions

In this work, we have quantified the desorption mechanism of DMMP from ZnPc layers by the evaluation of adsorption energy and with the TDS approach. We provide, for the first time, experimental evidence for the decomposition of DMMP as a result of desorption from ZnPc surfaces and we estimate the activation energies for the desorption of the decomposition products.

Our results show that DMMP molecules on the ZnPc surface undergo decomposition at temperatures above 500 K, producing formaldehydes, among other products. The desorption activation energy of the DMMP decomposition products is found to be higher than the DMMP adsorption energy calculated from theoretical modeling. We predicted this difference, as the molecule decomposition energy contributes to the total activation energy for desorption. Moreover, by supporting the experiment with semi-empirical simulations, we are able to compare desorption activation energies of species that resulted from DMMP decomposition with adsorption energies of those species on both the ZnPc and MoO_3_ substrates. We have demonstrated that the recorded desorption products are clearly a result of DMMP desorption from ZnPc and its successive decomposition.

Furthermore, we have estimated the expected changes in work function caused by the adsorption of DMMP and verified the sensing capability of the ZnPc layer, using impedance spectroscopy. This measurement demonstrated that the changes in electrical resistance and capacitance are consistent with the expected changes in the work function components. Finally, we have been able to demonstrate that ZnPc can be a potential sensor for the presence of sarin vapor in the air.

## Figures and Tables

**Table 1:** Desorption activation energies and relative areas of decomposed signals for species desorbing from unexposed and DMMP-exposed MoO3/ZnPc bilayers.

Desorbing Species	No	Unexposed MoO_3_/ZnPc			MoO_3_/ZnPc with DMMP		
		T, K	E_des_, eV	Rel. Area, %	T, K	E_des_, eV	Rel. Area, %
CH_2_O (30 amu)	1	-			538.1	1.73	3.3
	2				578.9	1.87	
					606.9	1.96	
	3				650.3	2.10	

H_2_ (2 amu)	1	-			516.9	1.66	15.9
					544.3	1.75	
	2	582.8	1.88	36.9	574.7	1.85	
		605.9	1.96		606.5	1.96	
		623.8	2.02				
	3	640.9	2.07		635.7	2.06	
		658.4	2.13		657.7	2.13	
	4	676.7	2.19		673.1	2.18	

CO/N_2_ (28 amu)	1	-			543.2	1.75	13.9
	2	-			574.8	1.85	
					609.3	1.97	
	3	655.7	2.12	9.5	637.7	2.06	
					655.8	2.12	
	4	675.6	2.19		-		

CO_2_/ H-C≡P (44 amu)	1	-			543.7	1.75	28.9
	2	583.6	1.88	18.9	578.2	1.86	
		606.2	1.96		607.1	1.96	
		617.3	1.99				
	3	643.3	2.08		639.7	2.06	
		660.5	2.14		661.6	2.14	
	4	673.8	2.18		-		

H_2_O (18 amu)	1	-			525.6	1.69	38.0
					545.0	1.75	
	2	582.9	1.88	34.8	572.0	1.84	
		612.9	1.98		588.0	1.90	
					608.4	1.96	
	3	641.5	2.07		638.4	2.07	
		657.2	2.13		651.8	2.11	
	4	675.9	2.19		-		

**Table 2:** Computed adsorption energies of the species desorbed from ZnPc on MoO3 substrate determined by semi-empirical PM6 method.

Adsorbed Species	E_ads_, eV (on ZnPc) from Theory	E_ads_, eV (on MoO_3_) from Theory	E^des^_min_, eV (Unexposed MoO_3_/ZnPc) from TDS	E^des^_min_, eV (MoO_3_/ZnPc with DMMP) from TDS
DMMP	0.8	2.5	-	-
CH_2_O	0.5	0.8	-	1.7
CO	0.9	3.4	2.1	1.7
CO_2_	0.1	0.4	1.9	1.7
PCH	1.8	1.9	-	1.7
H_2_O	0.4	0.7	1.9	1.7

## Data Availability

Not applicable.
